# Case report: Primary cytomegalovirus infection in a patient with late onset multiple sclerosis treated with dimethyl fumarate

**DOI:** 10.3389/fneur.2024.1363876

**Published:** 2024-10-30

**Authors:** Julia Sabin Muñoz, Alberto Díaz-De Santiago, José Cebrián Escudero, J. A. García Merino

**Affiliations:** ^1^Neuroimmunology Unit, Department of Neurology, University Hospital Puerta de Hierro Majadahonda, Madrid, Spain; ^2^Infectious Disease Unit, Department of Internal Medicine, University Hospital Puerta de Hierro Majadahonda, Madrid, Spain; ^3^Department of Neurology, University Hospital Rey Juan Carlos, Madrid, Spain

**Keywords:** late onset multiple sclerosis, immunosenescence, infection, safety, dimethyl fumarate

## Abstract

**Background:**

Treatment of multiple sclerosis (MS) with delayed-release dimethyl fumarate (DMF) has shown efficacy and safety in clinical trials. However, the occurrence of infectious complications, particularly in elderly patients, remains a concern.

**Methods:**

We present the case of a 63-year-old woman with late-onset MS treated with DMF, who developed a severe primary cytomegalovirus (CMV) infection. DMF was discontinued, and antiviral treatment was initiated, resulting in complete resolution of symptoms.

**Results:**

While DMF is generally considered safe in terms of opportunistic infections, this case highlights the possibility of serious infectious complications in eldery DMF-treated patients, even without documented lymphopenia.

**Conclusion:**

Advanced age and the associated immunological changes may contribute to an inadequate immune response to MS treatments, highlighting the importance of a careful treatment choice in elderly patients with MS. Further research and specific data on the safety and efficacy of DMF in this population are required to guide clinical decision-making.

## 1 Introduction

Multiple sclerosis (MS) is an inflammatory and demyelinating disease of the central nervous system (CNS). The drugs used in the MS treatment alter the functioning of the immune system cells through different mechanisms of action. Development of lymphopenia in relation to the use of disease modifying treatments (DMT) is one of the most frequent complications and has been classically associated with an increased risk of infection (1).

Delayed-release dimethyl fumarate (DMF) is an oral drug approved for relapsing–remitting MS in 2014. Although the exact mechanism of action is unknown, DMF has an anti-inflammatory effect. The drug reduces the proportion of T and B memory cells and increases the proportion of T and B naive cells. In addition to this, two signaling pathways have been proposed as possible mechanisms of action: the activation of the nuclear factor (erythroid-derived 2)-like 2 (Nrf2) antioxidant response pathway and the inhibition of NF-kB transcription factor. The Nrf2 transcriptional pathway is involved in maintaining cellular homeostasis, attenuating the production of proinflammatory cytokines, reducing the activation of macrophages, microglia, astrocytes, and promoting the restoration of blood–brain barrier integrity, thereby decreasing immune cell infiltration into the CNS. The NF-kB transcription factor plays a role in the regulation of pro-inflammatory genes and in the survival, activation, and differentiation of lymphocytes (2). Approximately 30% of patients trated with DMF develop lymphopenia between 6th and 12th months after the initiation of treatment but a decrease in absolute lymphocyte count (ALC) below 500/μl is observed only in 5% of cases (1, 3). Common risk factors associated with the development of lymphopenia due to DMT include age (4), the type and number of previous immunosuppressive treatments received (1), and baseline ALC before the onset of DMT. However, an increased risk of opportunistic infections has not been documented (3).

We present a late-onset MS patient treated with DMF, who was admitted due to general malaise, fever, and abdominal pain, being diagnosed with primary cytomegalovirus (CMV) infection.

## 2 Case description

A 63-year-old woman with a history of smoking and primary hypothyroidism under replacement therapy with a diagnosis of MS in April 2019. The initial symptoms were characterized by a right leg hypoesthesia that progressed over the following days to become bilateral and ascended to the thoracic region (level T5) and affected the upper limbs. There was no motor deficit or sphincteric involvement. An initial MRI was performed, confirming the presence of a demyelinating spinal cord lesion with gadolinium enhancement, and more than 10 demyelinating lesions located at the periventricular and infratentorial level. A lumbar puncture was performed, results for positive IgG oligoclonal bands. Other autoimmune and infectious pathologies were excluded by the corresponding serological test. In a follow-up MRI performed three months after this event, 3 new periventricular lesions was observed, confirming the diagnosis of MS.

The patient rejected an injectable medication. Due to some adverse prognostic factors (age and degree of inflammatory activity) and patient preference, we opted to iniciate oral treatment with DMF. Regular blood tests were conducted every 3 months during the first year of treatment, with no lymphopenia or alterations in renal or hepatic function. Annual MRI was stable. Since the patient started DMF, she remained with no evidence of disease activity (no relapses, no new MRI lesions and no disability progression), without symptoms for the daily life too. DMF was the first treatment after diagnosis, with no history of previous immunosuppression.

The patient was attended in the emergency department (ED) in June 2020 with severe abdominal pain, dyspepsia and nausea of more than 10 days duration. She discontinued the treatment with DMF 7 days before being admitted to the ED on medical advice, but she continued to get worse, adding fever up to 39°C, therefore, she decided to consult. Laboratory tests showed normal blood counts, including lymphocytes (2,57 ×10^3^/μL), but an altered liver profile with LDH 523 U/L (120-246 U/L), ALT 439 U/L (6–40 U/L), AST 325 U/L (6–40 U/L) and elevated CRP 14.90 mg/L (0,1-10 mg/L). No abnormal changes in urinalysis were observed. A PCR for SARS-COV2 in nasopharyngeal exudate was negative. Serology for CMV was positive for IgM, and negative for IgG, with a first PCR determination showing a value of 3,170 IU/ ml (log 3.5). Chest X-ray and abdominal ultrasound showed no pathological findings. HIV plasma viral load and serology were negative. Other infectious agents were ruled out (hepatitis A, B, C and E, Epstein Bar Virus, Q fever, parvovirus B19, syphilis, Human herpes virus 6 and 8, toxoplasmosis, Strongyloides).

**Figure 1:**
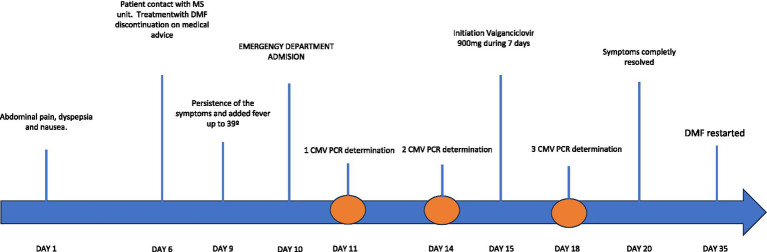
Timeline with clinical events of the primary CMV infection.

A CMV mononucleosis-like syndrome suspected and an initial conservative attitude adopted. However, the symptoms worsened and the fever persisted for 3 more days. A second PCR determination was carried out (11,400 IU / ml). It was also a worsening of the hepatic profile [LDH 331 U/L (120-246 U/L), ALT 537 U/L (6–40 U/L), AST 610 U/L (6–40 U/L)]. Therefore, we decided to initiate treatment with Valganciclovir 900 mg two times a day during 7 days. After 4 days of antiviral treatment, the PCR for CMV was negative (<35 UI/ml) and symptoms resolved completely, with normalization of laboratory tests. Fifteen days after hospital discharge, treatment with DMF was restarted with no further problems. We summarized the patients clinical events and laboratory results in Figure 1 and Table 1. The patient was followed up by the internal medicine department for one year, remaining stable. Simultaneously, the patient underwent biannual follow-up in the MS unit and remained stable too, without new infectious complications, and laboratory parameters within normal limits. Currently, she continues treatment with DMF at the moment.

**Table 1:** Laboratory data.

	CMV determination	Liver profile
Day 11	PCR 3170 copies/ml IgM positive IgG negative	LDH 523 U/L (120-246 U/L) ALT 439 U/L (6–40 U/L) AST 325 U/L (6–40 U/L) CRP 14.90 mg/L (0,1-10 mg/L)
Day 14	PCR 11400 copies/ml	LDH 331 U/L (120-246 U/L) ALT 537 U/L (6–40 U/L) AST 610 U/L (6–40 U/L)
Day 18	PCR	LDH 100 U/L (120-246 U/L) ALT 35 U/L (6–40 U/L) AST 10 U/L (6–40 U/L)

The concentration of IgG is expressed in IU/ml. A result is considered positive if it is >0.6 IU/mL. The concentration of serum IgM is expressed in AU/ml. A result is considered positive if it is >30 AU/mL. The samples were analyzed using the indirect chemiluminescence technique with automated analysis Liaison XL^®^. Cut off for PCR determination >35 copies/ml. The samples were analyzed by Cobas 6,800^®^ (Roche molecular systems).

## 3 Discussion

CMV is an enveloped double stranded DNA virus that belongs to the herpes virus family. Symptomatic infection is rare in immunocompetent hosts (sometimes causes a mild mononucleosis-like syndrome). Antiviral therapy is recommended only in cases of serious infections (primary or reactivation) of newborns, the elderly and immunosuppressed patients (5). The incidence of CMV complications associated with the different DMTs used in MS patients is rare (6). Cases of primary CMV infection and reactivation have been described with the use of Alemtuzumab, probably related to the marked depletion of T and B lymphocytes that occurs following the drug infusion (7).

To the best of our knowledge, this is the first reported case of a severe symptomatic CMV primary infection in a DMF-treated MS patient with no lymphopenia or other comorbidities. Symptomatic primary CMV infections usually affect patients with a severe degree of immunosuppression, but our patient was not initially within this group. However, the patient’s age and MS treatment with DMF should be considered as a risk factor for infection, so we should treat our patient as immunosuppressed.

There are no consistent data to consider DMF an immunosuppressive drug. In randomized clinical trials (RCTs), the use of DMF has been associated with a low risk of opportunistic infections. However, in recent years, some articles have highlighted this possibility (3). Up to 11 cases of CNS infections have been documented in patients treated with DMF with varying degrees of lymphopenia or even normal lymphocyte counts (8). Furthermore, some cases of progressive multifocal leukoencephalopathy have been documented, most of them related to prolonged low ALC (6). Studies show that DMF treatment induces a change in the immunophenotype in patients with early and prolonged treatment, resulting in a reduction of circulating central and peripheral memory T and B cells. An increase in the number of naïve T and B cells has also been described (9). Treatment with DMF involves a marked reduction in CD8 T cells compared to CD4 T cells (9, 10).

On the other hand, immunosenescence is considered a natural aging process that involves changes in the functioning of the innate and adaptive immune systems (involution of the thymus, decreased generation of naïve T cells, inverted CD4/CD8 ratio, alteration in the NK cell function and decreased tissue repair capacity) (11). This translates into a predisposition to carcinogenesis and an abnormal response to infectious agents and vaccines (12). In addition, age may alter the pharmacokinetics and pharmacodynamics of drugs (11). Due to the changes that age induces in the immune system, we could consider that this patient had an inadequate immune response, added to the effect of treatment with DMF, although lymphopenia was not documented at any time. The main limitation of this clinical case is that no samples were obtained for analysis of cell subpopulations.

Information on the characteristics of MS in older patients is limited. Most RCTs do not include patients over 55 years old. Therefore, we do not have sufficient data on the efficacy and safety of different DMTs in this population (12). Considering the effect of immunosenescence on the adaptative immune response, it leads us to suspect that treatment with DMF may predispose to the appearance of severe adverse effects in older MS patients (12). Advances in the treatment of MS and in the treatment of comorbidities have increased the life expectancy of our patients. In daily clinical practice we have more and more elderly patients who need DMTs (10). Therefore, special caution should be taken with their use in this subgroup of the population.

## 4 Conclusion

DMF is a drug with a good profile of efficacy, tolerability, and safety. Although RCTs have not shown a significant infectious risk even in patients undergoing long-term treatment, they have not provided data for elderly patients. In the absence of specific recommendations, we must treat older patients using the same algorithms as for younger ones. However, age can induce changes in the immune system that may predispose individuals to unexpected adverse events. This is an important factor to consider when choosing the most appropriate treatment for older patients.

## Data Availability

The datasets presented in this article are not readily available because of ethical and privacy restrictions. Requests to access the datasets should be directed to the corresponding author.
